# Automated landmark-based cat facial analysis and its applications

**DOI:** 10.3389/fvets.2024.1442634

**Published:** 2024-12-09

**Authors:** George Martvel, Teddy Lazebnik, Marcelo Feighelstein, Sebastian Meller, Ilan Shimshoni, Lauren Finka, Stelio P. L. Luna, Daniel S. Mills, Holger A. Volk, Anna Zamansky

**Affiliations:** ^1^Information Systems Department, University of Haifa, Haifa, Israel; ^2^Department of Mathematics, Ariel University, Ariel, Israel; ^3^Department of Small Animal Medicine and Surgery, University of Veterinary Medicine Hannover, Hannover, Germany; ^4^Cats Protection, National Cat Centre, Sussex, United Kingdom; ^5^School of Veterinary Medicine and Animal Science, São Paulo State University (Unesp), São Paulo, Brazil; ^6^Joseph Bank Laboratories, School of Life Sciences, University of Lincoln, Lincoln, United Kingdom

**Keywords:** artificial intelligence, facial landmark, cat behavior, cat morphology, pain assessment

## Abstract

Facial landmarks, widely studied in human affective computing, are beginning to gain interest in the animal domain. Specifically, landmark-based geometric morphometric methods have been used to objectively assess facial expressions in cats, focusing on pain recognition and the impact of breed-specific morphology on facial signaling. These methods employed a 48-landmark scheme grounded in cat facial anatomy. Manually annotating these landmarks, however, is a labor-intensive process, deeming it impractical for generating sufficiently large amounts of data for machine learning purposes and for use in applied real-time contexts with cats. Our previous work introduced an AI pipeline for automated landmark detection, which showed good performance in standard machine learning metrics. Nonetheless, the effectiveness of fully automated, end-to-end landmark-based systems for practical cat facial analysis tasks remained underexplored. In this paper we develop AI pipelines for three benchmark tasks using two previously collected datasets of cat faces. The tasks include automated cat breed recognition, cephalic type recognition and pain recognition. Our fully automated end-to-end pipelines reached accuracy of 75% and 66% in cephalic type and pain recognition respectively, suggesting that landmark-based approaches hold promise for automated pain assessment and morphological explorations.

## 1 Introduction

Facial expressions are universally acknowledged as key indicators of emotional states in mammals (1, 2). The link between facial expressions and emotions in humans has received considerable attention in research (3, 4). All mammals are known to display facial expressions (5). Analogously to humans, they are believed to communicate emotional states. This leads to the increase in interest in the study of facial expressions in the context of animal emotion and welfare studies (6–9).

Facial expressions and behavior also form an important part of animals' non-verbal communication. As such, it's essential that they are easily noticeable and interpretable by the receiver, implying that these signals must possess a degree of universality in their display. Yet, morphological characteristics in domesticated, and specifically in companion animals, may greatly vary due to selective breeding by humans (10–12), potentially affecting the distinctness of their facial expressions (13). However, the impact of this morphological diversity on the visual clarity of expressions and other forms of social signaling, as well as on the behavior and communication of companion animals, remains largely unstudied.

Although our understanding of facial visual signals, such as those caused by facial expressions and their relationship to animals' internal states, is quite limited, there has been some advancement mainly focusing on pain expressions. One of the most prevalent methods for pain assessment involves scoring by trained human experts. Species-specific pain assessment tools, known as grimace scales, focus on changes in an animal's facial features, and together with behavioral pain scales, they have been developed and validated for nearly all commonly domesticated species (with the notable exception of the dog, given their exceptional facial morphological diversity). Originally developed for rodents, these grimace scales have since been adapted for a range of mammalian species, including rats (14), rabbits (15), horses (16), pigs (17), ferrets (18), sheep (19, 20), and cats (21, 22).

In the context of studying facial appearance and visual signals, domestic cats are a particularly interesting exemplar in several aspects. First of all, cats display a wide variety of breed types and morphological features (although not as extreme as dogs), including a wide range of head shapes, from brachycephalic (e.g., the Persian) to dolichocephalic (e.g., the Siamese) (23). Additionally, the color of the coat, markings, and length of the fur are other elements that could influence the evaluation of facial expression in animals (24, 25), which potentially influence the ability to detect changes in facial expression in cats. Thus, they are a good initial exemplar for tackling the challenge of facial morphological diversity, which will need to be addressed for the more widespread use of AI in facial processing in animals. At the species level, domestic cats also show a diverse array of facial expressions, the majority of which have been systematically captured by the Cat Facial Action Coding System (CatFACS), linked to facial muscles (26). Changes in cats' facial shape have been linked to effective states such as fear, frustration, relaxed engagement, and pain (21, 22, 27). The available methods for cat pain assessment include three validated scales: the UNESP-Botucatu multidimensional composite pain scale (MCPS) (28), the Glasgow composite measure pain scale (CMPS) (29) and the Feline Grimace Scale (FGS) (22). However, all of these methods rely on the subjective judgments of humans, which may influence their reliability and validity. This leads to the need for the development of more objective methods for scoring and assessing pain, which are less susceptible to human bias.

Geometric morphometric analysis is a powerful tool that has been explored in the context of quantifying cat facial shape changes (13, 30). It uses points (facial landmarks) positioned on objects as proxies for shape. The landmark coordinates reflect their reciprocal locations, with differences in such locations across objects measuring the amount of shape variation. For instance, Finka et al. (30) applied this approach to quantify cat facial shape changes associated with pain. Images of 29 domestic short-haired female cats undergoing ovariohysterectomy were manually annotated using 48 landmarks specifically chosen for their relationship with underlying facial musculature and their relevance to cat-specific facial action units. A significant relationship was found between pain-linked Principal Components related to facial shape variation and the UNESP-Botucatu MCPS tool (28). In a similar manner, this approach was extended by Finka et al. (13) to explore the impact of cat breed and cephalic type variation on the relative positioning of facial landmarks. Major variations in baseline facial landmark configurations were identified within a population of common domestic cat breeds and diverse cephalic shapes. Variations in relative landmark positions were evident at both the cephalic and breed levels and were identified across all facial regions, including the ears, eyes, cheeks, mouth, and nose. Furthermore, while facial landmarks were able to differentiate between “pain” and “no pain” facial features in images of domestic short-haired cats, the painful cats of this breed were not reliably different from the neutral faces of other breeds.

These findings, which demonstrate that the geometric cat face model contains important visual information relevant to pain but is also potentially susceptible to “noise” caused by breed and cephalic variation in baseline features, formed a starting point for using machine learning (ML) techniques for automated recognition of cat pain in our previous work (31, 32). For example, in (31), the 48 facial landmarks suggested in Finka et al. (13, 30) were used in an ML model for cat pain recognition reaching above 72% on the dataset of Finka et al. (30). As this dataset was limited to young, adult female cats of a single breed and submitted to only one type of postoperative pain condition, this approach was subsequently extended to a more morphologically diverse dataset in Feighelstein et al. (32), reaching an even higher accuracy of detection of 77%, using the cat face model landmarks.

These results further indicated that the scheme of the 48 landmarks from Finka et al. (30) can contain useful visual signals sufficient for accurate recognition of internal states such as pain (30, 32), or for comparative studies across cat breeds and cephalic types (13). Feighelstein et al. (32) presented another interesting application of the geometric cat face model for investigating the explainability of pain recognition models by looking at average heat per landmark to understand more informative areas of the cat face for the ML model in pain recognition.

However, the landmark method heavily relied on the time-consuming and labor-intensive manual annotation of landmarks: in the case of Martvel et al. (33), it took skilled and trained annotators over 5.5 min to annotate one facial image, and our wider experience indicates this is typical for this type of activity. Martvel et al. (33, 34), developed an automated detector for cat facial landmarks, having introduced the first available annotated dataset with 48 landmarks: the Cat Facial Landmarks in the Wild (CatFLW) dataset (34). The model for automated landmark localization is based on a convolutional neural networks model and uses a magnifying ensemble method. Its performance in terms of normalized mean error (NME) was comparable and, in some cases, outperformed other models of this type with respect to human facial landmark localization. However, the question of whether the developed detector is useful for practical applications of cat facial analysis and whether automated landmark detection is sensitive enough for such tasks has remained open.

This study systematically investigates automated landmark-based approaches using three benchmark challenges related to cat facial analysis, which have been previously explored in the literature (30–32): breed, cephalic type, and pain recognition. We utilized the two datasets from these earlier studies to assess the performance of various landmark-based automated pipelines on these tasks. Additionally, we examined how their performance was impacted when the precise but labor-intensive manual landmarking process was substituted with a fully automated detection system.

## 2 Methods

### 2.1 Datasets

The dataset relating to cat pain was collected previously under the ethical approvals of the Institutional Animal Research Ethical Committee of the FMVZ-UNESP-Botucatu (protocol number of 20/2008) and the University of Lincoln, (UID: CoSREC252) as per Finka et al. (30). The dataset relating to cat breeds was collected previously under the ethical approvals of the Institutional Animal Research Ethical Committee of the FMVZ-UNESP-Botucatu (protocol number 20/2008). The use of this dataset and the generation of the data were approved by the delegated authority of Nottingham Trent University, Research Ethics Committee, as per Finka et al. (13). The current protocol using these datasets was reviewed by the Ethical Committee of the University of Haifa, and no further approval was required. All experiments were performed in accordance with relevant guidelines and regulations.

**Figure 1:**
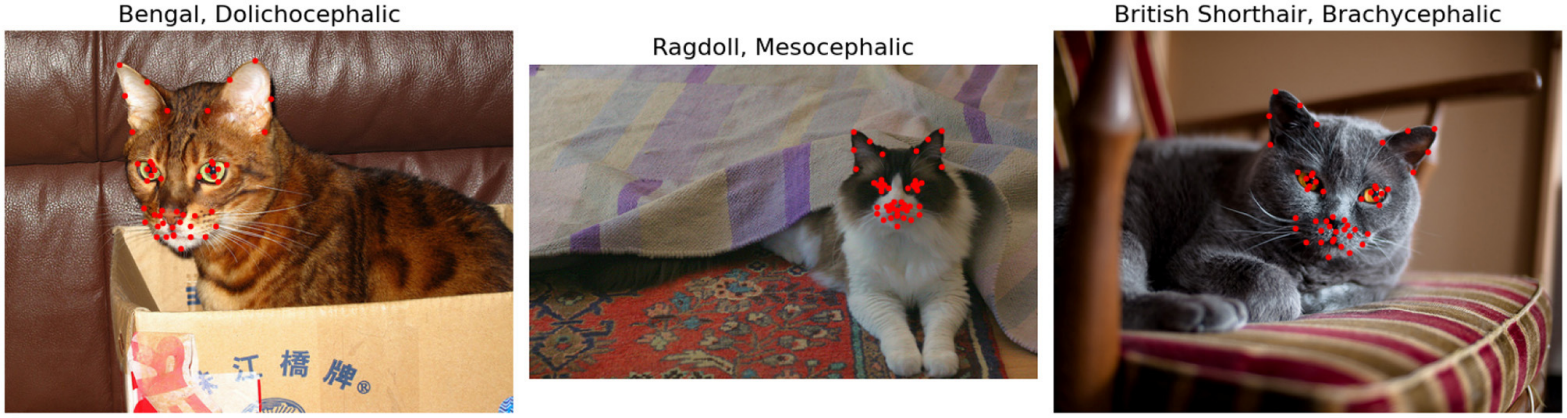
Cat images with 48 facial landmarks, breed, and cephalic type. Images are taken from the public Oxford IIIT Cats dataset (https://www.kaggle.com/datasets/imbikramsaha/cat-breeds).

The images in both datasets were manually annotated with 48 facial landmarks. Examples of images with the annotation structure are shown in Figure 1. Specific details of landmark placements and their relevance to facial musculature and CatFACS action units are provided in Finka et al. (13, 30). While images themselves were not augmented, the ground truth and detected landmarks underwent normalization and centering to enhance the robustness of subsequent analyses. Normalization scales the landmark coordinates so that they fall between 0 and 1, and centering translates the landmarks so that their centroid (mean position of all landmarks) coincides with the origin of the coordinate system. Additional augmentation techniques on data include shifting random landmarks, complete landmark shifting, and rotations.

#### 2.1.1 The *Cat Breed Dataset*

**Figure 2:**
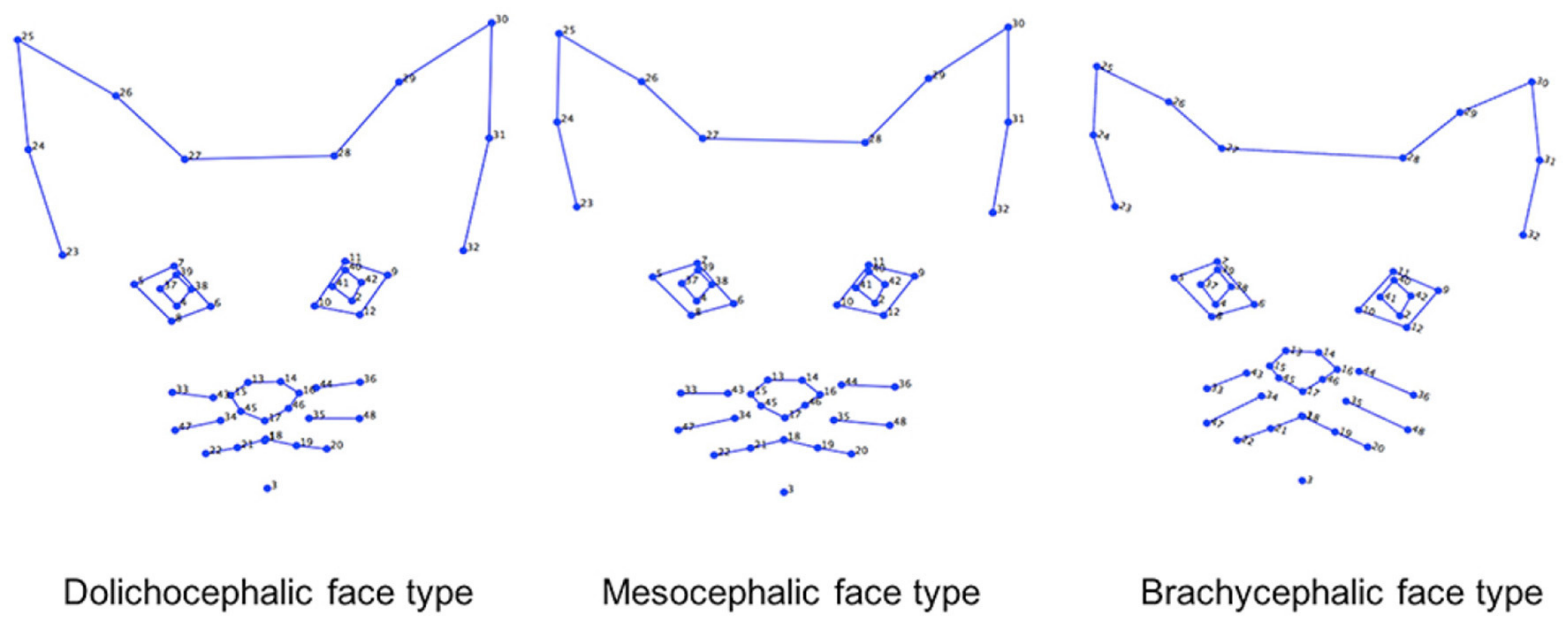
Average facial landmarks (annotated manually) for the three different cephalic types (13).

This dataset included 1,662 images of cat facial images annotated across *n* = 18 common breeds. The images were sourced from Oxford Pet Dataset (35) and Google images. The breeds were further divided by Finka et al. (13) into three categorical cephalic types: dolichocephalic, mesocephalic, and brachycephalic. Dolichocephalic, or “long-headed” cats, have elongated faces with lengths greater than the width. Mesocephalic, or “middle-headed” cats have square-like faces with approximately equal width and length of the face. Brachycephalic, or “short-headed” cats, have flat faces, and the muzzle area is located closer to the eyes. Figure 2 presents average landmark annotations for the three cephalic types. The distribution of images over the types and breeds is provided in Table 1.

**Table 1:** Number of samples for each cat cephalic type and breed.

**Cephalic type**	**Breed**	**Number of samples**
Dolichocephalic	Total	390
	Abyssinian	100
	Bengal	100
	Egyptian Mau	89
	Oriental Shorthair	101
Mesocephalic	Total	705
	Birman	100
	Domestic longhair	99
	Domestic shorthair	107
	Maine coon	101
	Norwegian forest Cat	88
	Ragdoll	110
	Russian blue	100
Brachycephalic	Total	567
	American shorthair	96
	Bombay	45
	Devon rex	96
	Exotic shorthair	100
	Persian	101
	Scottish fold	31
	British shorthair	98

#### 2.1.2 The *Cat Pain Dataset*

We used the dataset generated as part of a previous study by Finka et al. (30). The raw data comprised of footage from 29 healthy domestic short-haired female cats undergoing ovariohysterectomy as described in Brondani et al. (28). Cats were recorded at different time points corresponding to varying intensities of pain: pre-surgery (between 18–24 h during the preoperative period), 1-h post-surgery (between 30 min and 1 h after the end of the surgery, and prior to administration of additional analgesics), and post-rescue analgesia (approximately 4 h after postoperative analgesia). The final dataset contains images from 26 cat individuals with 232 images of “No Pain” (pre-surgery stage and post-rescue analgesia stage), and 232 images of “Pain” (1-h post surgery), overall 464 images.

### 2.2 The cat facial landmarks automated detector

The cat facial landmark detector used here is presented in Martvel et al. (33). This AI pipeline uses a magnifying method to localize landmarks, taking an image as input and producing 48 cats' facial landmarks. First, it localizes the face (so no preprocessing of the image is required), then determines five regions of interest (ears, eyes, and the whiskers area), and then localizes landmarks in each of these regions. The model was trained on the Cat Facial Landmarks in the Wild (CatFLW) dataset (34), which contains 2091 facial images of cats annotated with the 48 landmark scheme from Finka et al. (30).

### 2.3 Machine learning models

We have formulated the following benchmark tasks to be addressed:

1. *Cat breed recognition*: given a facial image of a cat, detect the cat's breed out of 18 classes (the full list of the 18 breeds is presented in Table 1).
2. *Cephalic type recognition*: given a facial image of a cat, detect its cephalic type out of 3 classes (dolichocephalic, mesocephalic or brachycephalic, see Table 1).
3. *Pain recognition*: given a facial image of a cat, detect whether it is in pain (binary “Pain”/“No pain” classification).

**Figure 3:**
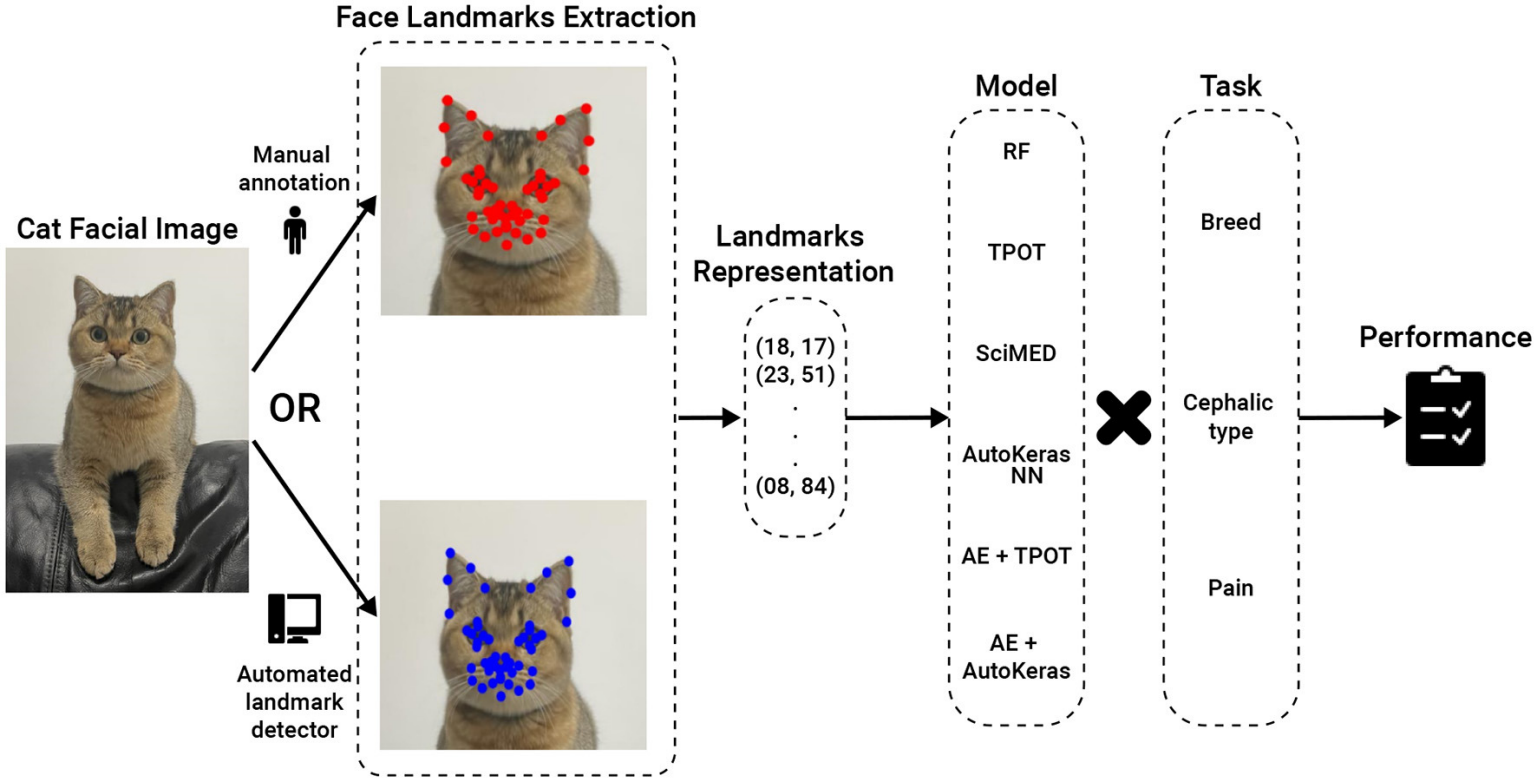
AI Pipeline Overview. The input is a cat facial image, which can be annotated either manually or using an automated landmark detector. The intermediate stage is 48 landmark coordinates, which are then fed to a machine learning model for classification. RF, Random Forest; TPOT, Tree-Based Pipeline Optimization Tool; SciMed, Scientist-Machine Equation Detector; NN, neural network; AE, AutoEncoder.

Figure 3 presents a high-level overview of the AI pipelines studied.

For the first two tasks, the models were trained using the *Cat Breed Dataset*. We tested a sequence of ML models with increasing levels of complexity. Each model was used for both the breed and cephalic type recognition classification tasks as the input features are identical, and only the target features are altered. Initially, we tested the Random Forest (RF) model (36) with a grid-search hyperparameter tuning (picked manually) (37) and bidirectional elimination for the feature selection (38). Specifically, we used a mixture of cost-complexity and SAT-based post-pruning on the trees in the RF model to obtain better generalization (39, 40). Afterward, we tested the Tree-Based Pipeline Optimization Tool (TPOT), a genetic algorithm-based automatic ML library (41). TPOT produces a full ML pipeline, including feature selection engineering, model selection, model ensemble, and hyperparameter tuning. In an orthogonal testing direction, we used the Scientist-Machine Equation Detector (SciMED) symbolic regression model, which searches for an analytical function between the input features and the target feature (42). Next, we moved to the deep-learning realm as it is known to be able to handle high-dimensional and complex tasks (43–45), as the ones we deal with. To this end, we initially developed our own neural network (NN) model using a manual trial-and-error process. Namely, we obtain a feed-forward (i.e., fully connected) NN with three layers (96, 64, 32, and 18), separated by dropout layers with *p* = 0.1 drop rate. The Adam optimizer with a learning rate of 10^−4^ is used for the training procedure with a batch size of 8. The cross-entropy loss was utilized given the categorical nature of the problem, and the primary metric for model evaluation during training was accuracy. To test a larger-scale NN architecture scale, we tested AutoKeras (46), an automatic deep-learning library that automatically searches for NN architectures and training hyper-parameters. Following this promising direction, we came to the conclusion that the input feature space is not informative as the data represents locations in an image (in a Cartesian coordinate system). As such, we decided to divide the classification and input data representation tasks. We repeated the TPOT and AutoKeras tests such that both were obtained as an input a 16-dimensional input generated from a fully connected AutoEncoder NN (47) (AE) with three layers for the encoder and decoder parts (96, 64, 32, 16, 32, 64, and 96).

The third task was previously explored on the *Cat Pain Dataset* in Feighelstein et al. (31), where manually annotated facial landmarks were used. We aimed to study an end-to-end automated pipeline, focusing specifically on how the pain recognition model performance is affected when we replace manually annotated landmarks with automatically detected ones. For a fair comparison, we followed the same preprocessing scheme as Feighelstein et al. (31). Namely, a preprocessing pipeline took as input images annotated with 48 landmarks, produced a set of multi-region vectors, and then introduced them into the classification model. During the preprocessing phase, the landmarks were centered as part of face alignment and vectorized based on the four facial regions. We used the same structured, fully connected NN for the classification as in the breed/type classification. The model is trained during ten epochs, optimizing a cross-entropy loss function using an Adam optimizer with a 0.1 learning rate and a batch size of 32. On each epoch, the training set is normalized using standard scaling and augmented. We again chose the model's hyperparameters that achieved the best (minimal) validation loss.

As a validation method for the first and second tasks (carried out on the *Cat Breed Dataset*), we used 5-fold cross-validation. For the third task, which was carried out in a different (balanced) *Cat Pain Dataset*, we used the stricter leave-one-subject-out cross-validation with no subject overlap (48). Due to the relatively low numbers of cats (*n* = 27 after dataset balancing) in the dataset, following this method is more appropriate (8, 49). By separating the subjects used for training, validation, and testing, respectively, we enforce generalization to unseen subjects and ensure that no specific features of an individual are used for classification. For evaluation of the facial landmark detector performance, the normalized mean error (NME) (50) and normalized root mean squared error (NRMSE) metrics using the inter-ocular distance (the distance between the outer corners of the eyes) were used to measure average detector errors. Additionally, to inspect how well breeds and cephalic types are separated when using manual vs. automatically detected landmarks, we used the t-distributed stochastic neighbor embedding (t-SNE) (51) transformation to visualize the high-dimensional data in two dimensions. This transformation provides a visual and more intuitive representation of the model's learned feature space. Finally, each of the models was tested with manually annotated landmarks and with automatically detected landmarks using the detection pipeline from (33) described above. The model's performance was measured using accuracy and *F*_1_-score.

## 3 Results

### 3.1 Landmark detection

Table 2 presents the average normalized mean error (NME) and normalized root mean squared error (NRMSE) measurements on the automatic detection of facial landmarks as compared to manual annotations. The largest error is observed for the dolichocephalic breeds and the smallest—for the mesocephalic ones. In terms of breeds, the “easiest” breed (with minimal error) for landmark detection is the (brachycephalic) British Shorthair, and the “hardest” breed (with maximal error) is the (dolichocephalic) Oriental Shorthair.

**Table 2:** Average normalized mean error (NME) and normalized root mean squared error (NRMSE) for different cephalic types and breeds (in %).

**Cephalic type**	**Breed**	**NME (%)**	**NRMSE (%)**
Dolichocephalic	Type average	13.49	14.02
	Abyssinian	9.49	11.20
	Bengal	9.78	10.99
	Egyptian Mau	9.55	11.73
	Oriental shorthair	25.92	22.76
Mesocephalic	Type average	9.16	9.89
	Birman	9.56	10.12
	Domestic longhair	8.34	8.86
	Domestic shorthair	8.24	9.29
	Maine coon	10.03	10.69
	Norwegian forest cat	9.95	10.84
	Ragdoll	9.58	10.12
	Russian blue	8.57	9.46
Brachycephalic	Type average	11.27	11.07
	American shorthair	8.82	9.30
	Bombay	9.93	10.57
	Devon rex	20.68	18.79
	Exotic shorthair	10.11	9.30
	Persian	10.92	10.38
	Scottish fold	10.59	10.81
	British shorthair	7.87	8.18

### 3.2 Breed and cephalic type recognition

**Figure 4:**
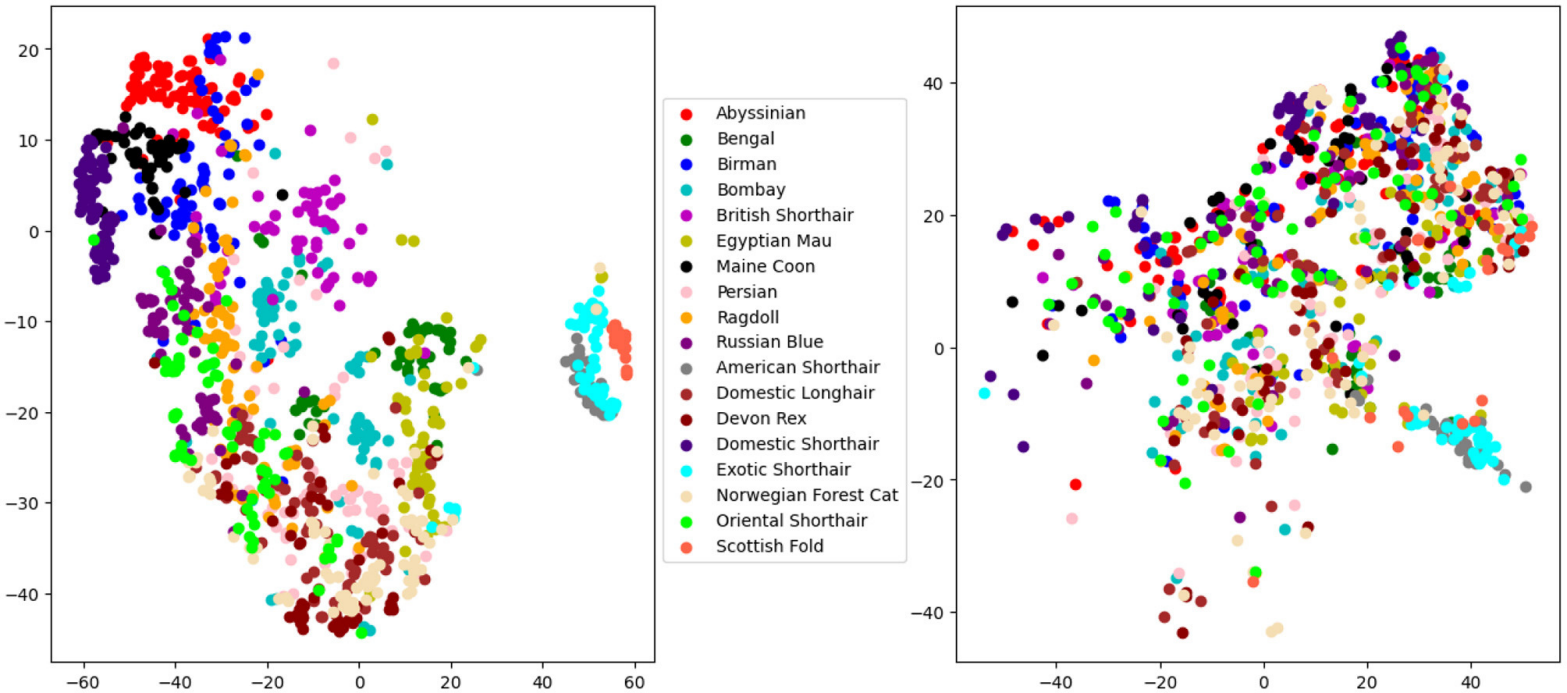
T-distributed stochastic neighbor embedding (t-SNE) distributions for breed recognition: manual landmarks **(left)** and automatically detected landmarks **(right)**.

**Figure 5:**
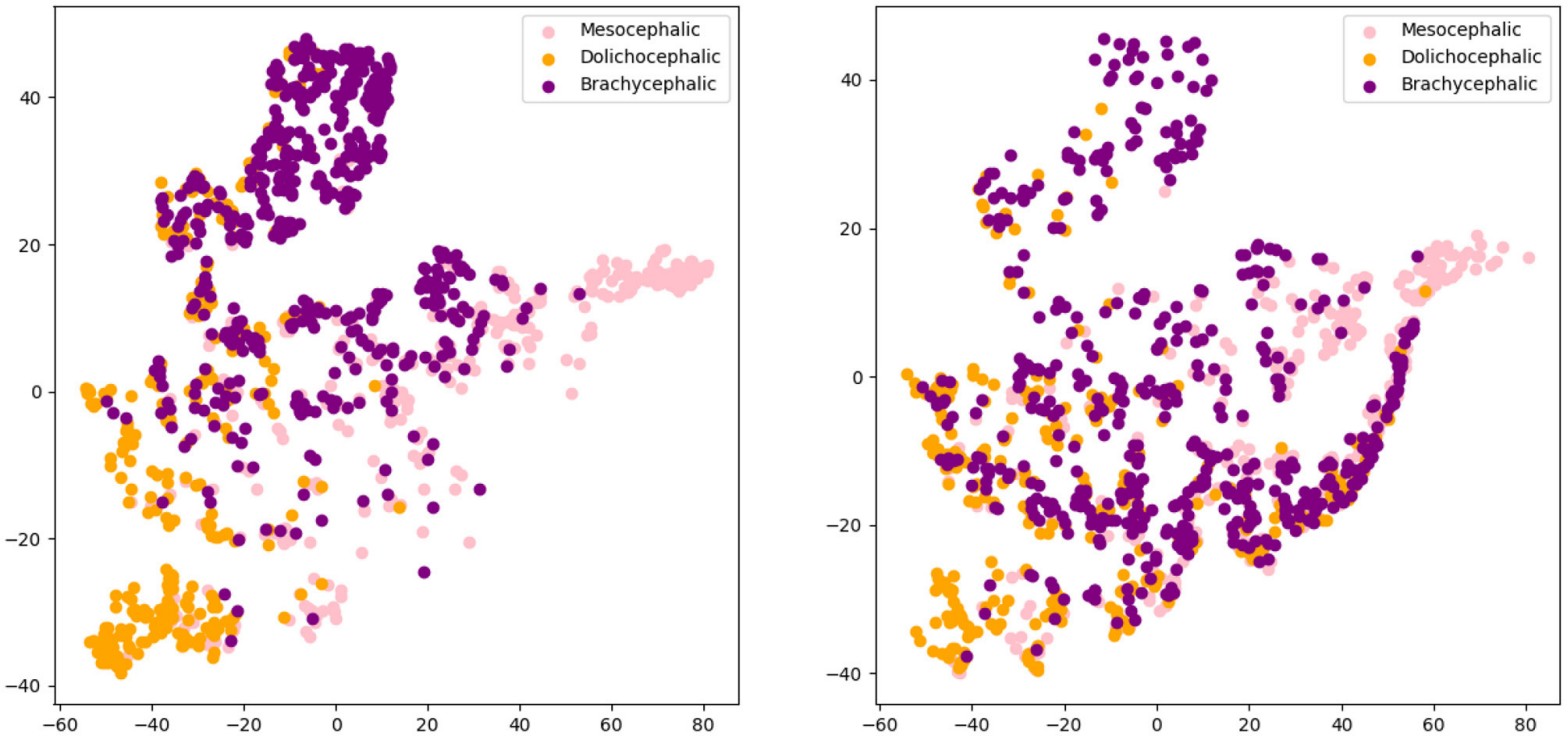
T-distributed stochastic neighbor embedding (t-SNE) distributions for cephalic type recognition: manual landmarks **(left)** and automatically detected landmarks **(right)**.

Figures 4, 5 present the t-SNE visualization of the 18 breeds and 3 cephalic types, respectively, using both manual and automatically detected landmarks. The visualization shows the possibility for a better potential separation between breeds and types when using the former; it also shows a better separation between cephalic types than between breeds when using automatically detected landmarks.

Table 3 presents a comparison of the performance of pipelines in the first two tasks, using different architectures, with the best being AE+AutoKeras in both. Tables 4, 5 present the metrics in a break-down to breeds and types using AE+AutoKeras model.

**Table 3:** Comparison of model performance for manual and automatic landmarks for the tasks of cephalic type and breed recognition.

**Task**	**Model**	**Manual**		**Automatic**	
		**Accuracy**	*F*_1_ **-score**	**Accuracy**	*F*_1_ **-score**
Cephalic type	RF	0.72	0.71	0.66	0.64
	TPOT	0.78	0.76	0.73	0.70
	SciMED	0.59	0.61	0.54	0.58
	NN	0.82	0.79	0.78	0.75
	AutoKeras	0.82	0.80	0.79	0.75
	AE + TPOT	0.82	0.80	0.79	0.74
	AE + AutoKeras	0.82	0.80	0.79	0.75
Breed	RF	0.54	0.52	0.38	0.34
	TPOT	0.62	0.61	0.43	0.42
	SciMED	0.55	0.55	0.42	0.40
	NN	0.68	0.67	0.47	0.45
	AutoKeras	0.69	0.67	0.48	0.46
	AE + TPOT	0.70	0.68	0.47	0.44
	AE + AutoKeras	0.70	0.68	0.48	0.45

RF, Random Forest; TPOT, Tree-Based Pipeline Optimization Tool; SciMed, Scientist-Machine Equation Detector; NN, neural network; AE, AutoEncoder.

**Table 4:** Accuracy and *F*_1_-score metrics for various breeds using AE+AutoKeras model.

**Breed**	**Manual**		**Automatic**	
	**Accuracy**	*F*_1_ **-score**	**Accuracy**	*F*_1_ **-score**
Abyssinian	0.86	0.86	0.63	0.58
Bengal	0.51	0.48	0.42	0.41
Birman	0.60	0.57	0.37	0.33
Bombay	0.78	0.77	0.50	0.50
British shorthair	0.69	0.65	0.51	0.45
Egyptian Mau	0.74	0.72	0.54	0.52
Maine coon	0.92	0.91	0.63	0.60
Persian	0.51	0.51	0.38	0.36
Ragdoll	0.67	0.64	0.43	0.41
Russian blue	0.56	0.53	0.41	0.37
American shorthair	0.94	0.94	0.58	0.54
Domestic longhair	0.61	0.58	0.40	0.39
Devon rex	0.52	0.49	0.34	0.30
Domestic shorthair	0.89	0.86	0.61	0.57
Exotic shorthair	0.90	0.87	0.65	0.63
Norwegian forest cat	0.52	0.50	0.40	0.37
Oriental shorthair	0.54	0.53	0.34	0.30
Scottish fold	0.87	0.86	0.55	0.50
**Average**	0.70	0.68	0.48	0.45

**Table 5:** Accuracy and *F*_1_-score metrics for various cephalic types.

	**Manual**		**Automatic**	
	**Accuracy**	*F*_1_ **-score**	**Accuracy**	*F*_1_ **-score**
Dolichocephalic	0.79	0.78	0.78	0.76
Mesocephalic	0.81	0.79	0.78	0.73
Brachycephalic	0.86	0.83	0.81	0.77
**Average**	0.82	0.80	0.79	0.75

### 3.3 Pain recognition

Table 6 presents the performance of the pain recognition pipeline for manual and automatically detected landmarks. Performance for the latter is lower, reaching only 66% accuracy, with the former performing with 73%.

**Table 6:** Pain recognition model performance metrics.

**Manual**		**Automatic**	
**Accuracy**	*F*_1_ **-score**	**Accuracy**	*F*_1_ **-score**
0.73	0.76	0.66	0.67

## 4 Discussion

The facial landmark scheme of 48 landmarks, systematically developed based on cat facial anatomy in Finka et al. (30), has demonstrated its utility in several applications. That includes geometric morphometric methods for accurately quantifying changes in cat facial features (13, 30), landmark-based AI models for pain recognition (31, 32) and enhancing the explainability of deep learning models related to facial analysis of cats (32).

The annotation process is, however, extremely laborious and time-consuming. According to Martvel et al. (33), it takes skilled and trained annotators over 5.5 min to annotate each image. For the *Cat Breed Dataset* alone, this translates to more than 161 hours of manual annotation work for a relatively small dataset, underscoring the pressing need for automation of this process.

Automated localization of facial landmarks, also known as fiducial points (52), is a cornerstone of the field of automated human facial analysis. It has numerous applications for face alignment, feature extraction, facial expression recognition, head pose estimation, eye gaze tracking, facial unit recognition, and many more tasks (53–57); which has been addressed by a growing body of work. In the human domain, affective computing is a well-developed discipline, integrating aspects of facial expression analysis and gesture recognition with advanced, real-time emotion recognition platforms such as Noldus Face Reader, Microsoft Azure Cognitive Services, Affectiva AFFDEX, and Emotient FACET. Moreover, AI automatic pain estimation applications for assessing human pain from facial expressions have already been integrated into clinical settings for non-verbal patients. An example is PainChek (58), which uses facial landmarking techniques and has already been applied for patients with dementia and infants (59, 60).

The question arises: how close are we to developing mobile applications like “CatPainChek” or “Cat Google Translate” that can accurately interpret cats' affective states? To address this, it's crucial to scrutinize the methodologies used in creating AI models for recognizing human affective states. Human face and gesture analysis has existed as a research area since the 1970s, and a vast number of datasets and manual annotations of facial expressions and emotional states were available to boost the development of AI algorithms and models. These datasets contained annotations of millions of frames and, in many cases, were created using actors. For instance, the Actor Study Database, published in Seuss et al. (61), contains 68 minutes of high-quality videos of facial expressions performed by 21 actors, whose tasks ranged from displaying specific Action Units and their combinations at different intensities to enactment of a variety of emotion scenarios. Clearly, compiling datasets of cat body language and facial expressions tied to their emotional and welfare states is significantly more complex than it is for humans since cats obviously cannot serve as “actors” nor can we be totally confident about their emotional state, although scientific frameworks for inferring different emotional states in non-human animals are being developed (62). These include whole subject and contextual evaluation in addition to facial analysis. Indeed, pain is perhaps comparatively the easier affective state that we can both ethically and practically operationalize with certainty compared to other, more complex feline emotional states.

The development of automated facial analysis techniques for animals is just beginning to emerge. Broomé et al. (49) review state-of-the-art studies in the field. Critical to this is the need for valid benchmarks, which are commonly available in human domains. In machine learning research, benchmarks typically consist of well-defined datasets, evaluation metrics, and specific tasks or challenges. Benchmarks are crucial in research as they facilitate the objective comparison of different approaches, promoting transparency and reproducibility in scientific findings. For instance, in the human domain, there are numerous benchmark datasets [such as the Cohn-Kanade dataset (63), the Toronto face database (64), the Actor Study Database (61) and many more]. This lack of similar datasets for animals is a significant hindrance to progress in the field but is not insurmountable with appropriate investment. Species-specific benchmarking resources can promote comparison between approaches and systematize the field. Another issue discussed in Broome et al. is considerations of ethics and privacy, especially when producing datasets with animal participants where emotional states and especially pain are induced. This often makes it difficult to make datasets publicly accessible.

The contributions of the current study address this gap in several dimensions. First of all, we evaluated the usefulness of the automated detector of cat facial landmarks on three non-trivial machine learning tasks: breed, cephalic type, and pain recognition. As anticipated, substituting manually identified landmarks with their automatically detected equivalents led to a decrease in performance across all tasks. The key question is to what extent this reduction in accuracy is an acceptable trade-off for achieving complete automation. The pain recognition task presented a 7% drop in accuracy and 9% drop in *F*_1_-score. The cephalic type task presented only 5% drop in *F*_1_-score for the best model. The breed recognition task turned out to be the most sensitive in this context, showing a drop in 23% in *F*_1_-score for the best model. It should be noted that since the type and breed recognition tasks used unbalanced datasets, *F*_1_-score is the more informative metric for performance in this case.

**Figure 6:**
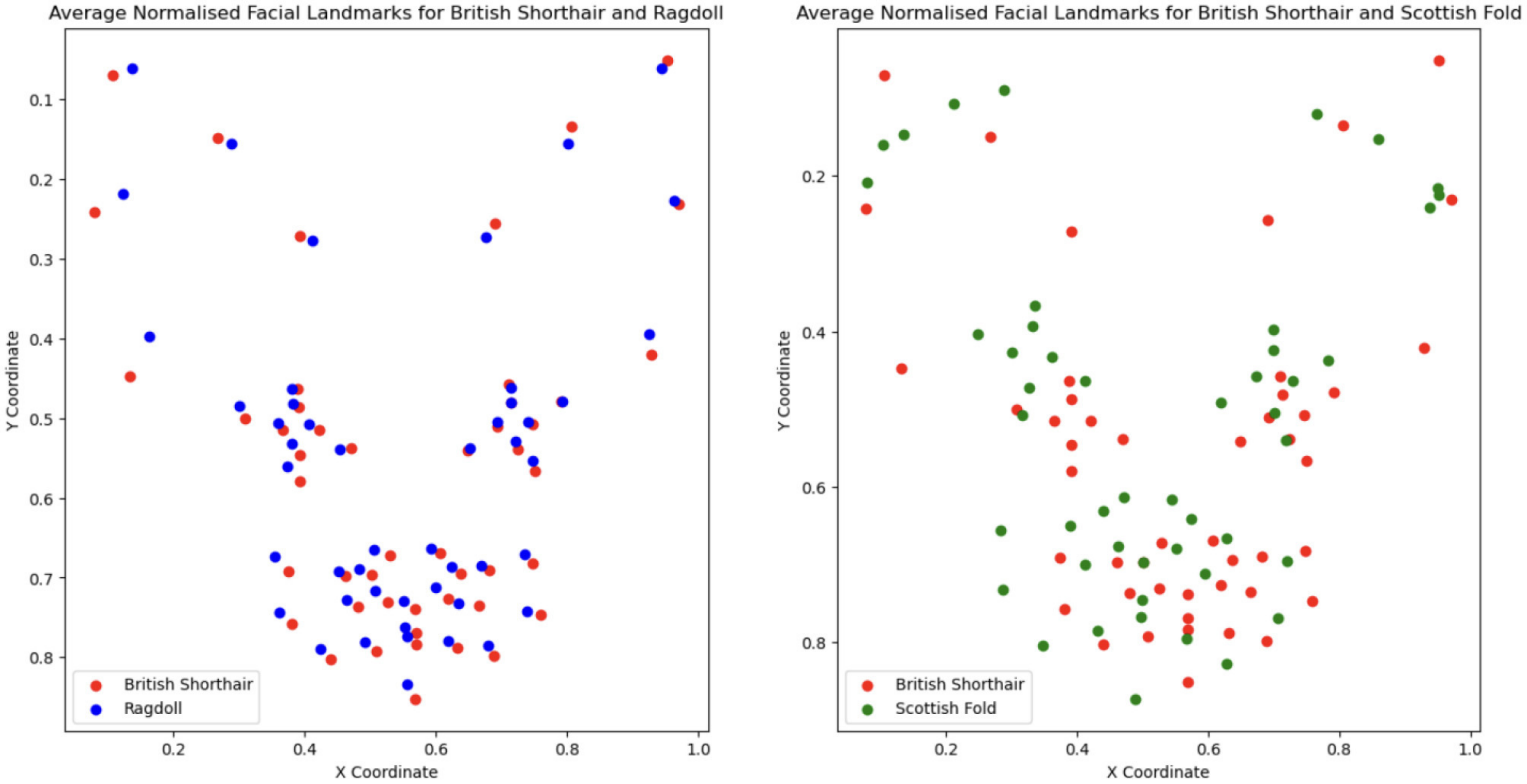
Comparison of breed-averaged normalized manual facial landmarks for British Shorthair, Ragdoll, and Scottish Fold.

Going deeper into the break-down to breeds, Table 4 shows that the largest drop in performance (moving from manual to automatic) is observed in Americal Short Hair (40%), Scottish Fold (36%) and Maine Coon (31%). The smallest drop occurs in Bengal (7%); however, the performance of the model is very low for both. The breed recognition task turns out to be extremely difficult for landmark-based models even when using manual landmarks: the best model reaches 68% in *F*_1_-score. As illustrated in Figure 6, this difficulty arises from the subtle differences in facial landmarks within certain breeds like the British Shorthair and Ragdoll. Despite belonging to distinct cephalic types, these breeds exhibit very similar geometric structures [this could be due to the two-dimensional geometric information not sufficiently factoring in muzzle length; it could also be introduced by labeling “errors” due to the breed and type labeling methodology used in Finka et al. (13)]. Other breeds, however, such as the Scottish Fold and Maine Coon, have distinct visual characteristics that set them apart, contributing to the relatively higher accuracy observed in their classification. Landmark-based approaches seem not to be the optimal choice for breed classification. Moving to black-box models has the price of losing explainability; however, such approaches seem more promising for this type of task. For instance, Ráduly et al. (65) reach very high accuracy in dog breed classification using deep learning techniques (although their dataset is much larger than the one used in this study).

Our models are much more successful with the cephalic type classification task, on the other hand, which presents small differences between manual and automated landmarks and has a high performance of 80% in *F*_1_-score. Scrutinizing the breakdown to the types on Table 5, the best performance is for the brachycephalic type, which also exhibits the largest drop in accuracy when moving to automated landmarks. This could be explained by the illustration in Figure 2, which shows the brachycephalic average landmarks have the most distinctive structure out of the three types.

The considerable differences in performance both in landmark detection and classification among various breeds (even within one cephalic type) and cephalic types can potentially be partly attributed to the unbalanced representation of each category in our existing datasets. Based on the results of Sexton et al. (24), we can also assume that the color of the coat and markings are other elements that influence the evaluation of landmark detection. It's plausible that training our automated detector on specific cephalic types or breeds could enhance its accuracy for those particular groups. However, the cat pain datasets currently available lack a diverse range of breeds and cephalic types. A valuable avenue for future research would be to enlarge these datasets and investigate the potential of models tailored to specific breeds and types within “pain” and “no pain” classifications.

This study has created landmark-based *benchmark* challenges for cat facial analysis by providing datasets, annotations, and benchmark models as a point of reference. Our aim is for these benchmarks to act as a foundational resource, enabling researchers to improve these results and develop new methods for analyzing cat facial expressions. In particular, it is important to highlight the difficulty in landmark detection of dolichocephalic breeds such as the Oriental short hair. The morphological traits of these breeds are quite extreme, and future datasets should aim to include more samples from these breeds.

In this context, another recent study by Steagall et al. (66) should be mentioned, which provided a deep learning pipeline for cat pain recognition based on facial landmarks. The study used an alternative reduced scheme of 37 landmarks, developing also an automated detector for them. Their pipeline is significantly outperformed by the current pipeline in terms of landmarks detection accuracy (their pipeline reaching only 16.76% NRMSE at best on aligned faces and worse on non-aligned, compared to the results in Table 2 on non-aligned images), while using fewer landmarks. However, it further highlights the utility of automated cat facial landmark detection and the importance of systematizing this emerging field. It is important to recognize, furthermore, that the creation of multiple landmark schemes that are difficult to compare poses a significant challenge to the systematic progression of the field. Future advancements in landmarking techniques should aim to build upon existing frameworks, leveraging the progress already made in this area.

## 5 Conclusions

In this paper we systematically explored the usefulness of the automated cat facial landmark detector introduced in Martvel et al. (33) for three tasks related to facial analysis: breed, cephalic type and pain recognition. The breed recognition pipeline performed below chance level, indicating that deep learning approaches are a better fit for this task. Our fully automated end-to-end pipelines reached accuracy of 75% and 66% in cephalic type and pain recognition respectively, suggesting that landmark-based approaches hold promise for automated pain assessment and morphological explorations.

## Data Availability

The raw data supporting the conclusions of this article will be made available by the authors, without undue reservation.
